# Insights into the Understanding of Adsorption Behaviors of Legacy and Emerging Per- and Polyfluoroalkyl Substances (PFASs) on Various Anion-Exchange Resins

**DOI:** 10.3390/toxics11020161

**Published:** 2023-02-09

**Authors:** Hong-Ming Tan, Chang-Gui Pan, Chao Yin, Kefu Yu

**Affiliations:** 1 Guangxi Laboratory on the Study of Coral Reefs in the South China Sea, School of Marine Sciences, Guangxi University, Nanning 530004, China; 2 School of Resources, Environment and Materials, Guangxi University, Nanning 530004, China; 3 Southern Marine Science and Engineering Guangdong Laboratory, Zhuhai 519000, China

**Keywords:** emerging PFASs, ion exchange, adsorption competition, solution chemistry

## Abstract

Per- and polyfluoroalkyl substances (PFASs) have received extensive attention due to their various harmful effects. In this study, the adsorptive removal of 10 legacy and emerging PFASs by four anion-exchange resins (including gel and macroreticular resins) were systematically investigated. Our results showed that the capacities of resins absorbing PFASs were ranked in the following order: gel strong base HPR4700 (297~300 μg/g) ≈ macroreticular strong base S6368 (294~300 μg/g) ≈ macroreticular weak base A111S (289~300 μg/g) > gel weak base WA10 (233~297 μg/g). Adsorption kinetic results indicated that the adsorption process might involve chemical and Henry regime adsorption or reaction control. Intraparticle diffusion was probably the major removal step. Co-existing fulvic acid (0.5, 1, 5 mg/L) and inorganic anions (5 mg/L of sulfate, carbonate, bicarbonate) would hinder the PFAS removal by resins with WA10 showing the highest inhibition rate of 17% and 71%, respectively. The adsorption capacities of PFBA decreased from 233 μg/g to 194 μg/g, and from 233 μg/g to 67 μg/g in the presence of fulvic acid and inorganic anions, respectively. PFASs were more easily removed by HPR4700, S6368, and A111S under neutral and alkaline environment. Moreover, WA10 was not able to remove PFASs under an alkaline medium. This study offered theoretical support for removing PFASs from aqueous phases with various resins.

## 1. Introduction

Per- and polyfluoroalkyl substances (PFASs) are synthetic organic compounds which are widely used in various industrial and commercial applications [1,2]. Legacy PFASs can repel both water and oil due to the soluble anionic end group and nonpolar perfluoroalkyl backbone [3]. Wide application of PFAS has resulted in the ubiquitous presence of these chemicals in water [4], sediment [5], sludge [6], atmosphere [7], and elsewhere. Because of the high bond energy of the C-F covalent bond (approximately 466 kJ/mol), several PFASs are extremely persistent in the environment and show strong resistance to biological or chemical degradation [8,9]. Consequently, they can be biomagnified along the trophic level and show toxicity to the brain, blood, kidney, liver, and other tissues [10,11]. Therefore, the most well-known PFASs, perfluorooctane sulfonic acid (PFOS) and perfluorooctanoic acid (PFOA), were listed as persistent organic pollutants (POPs) under the Stockholm Convention in 2009 and 2019 [12,13]. Correspondingly, several PFAS substitutes were introduced into the market [14,15]. For instance, hexafluoropropylene oxide dimer acid (GenX) and 6:2 fluorotelomer sulfonate (6:2 FTSA) are typical representatives of emerging alternatives [16,17]. However, such alternatives were also widely detected in various matrices [18].

Adsorption has been widely applied in the removal of PFASs from water [19]. As PFASs mainly exist as anions in the aqueous phase, anion exchange may be appropriate for their removal [20,21]. For example, IRA67 resin exhibited high adsorption capacities for both F-53B (4.2 mmol/g) and PFOS (5.5 mmol/g) [22]. Likewise, it has been shown that the adsorption of PFOS onto weak base resins achieved 4–5 mmol/g, with polyacrylic resin exhibiting higher removal than polystyrene resin [23,24]. Moreover, Zaggia et al. (2016) found that A600E with weak hydrophobicity showed a lower adsorption capacity as compared to strongly hydrophobic A532E for the removal of legacy PFASs from drinking water [25]. It has also been shown that the adsorption of short-chain PFAS onto resins might be more rapid than long-chain PFAS, suggesting that the perfluoroalkyl tail length can influence the adsorption kinetics [23,26]. However, another study found that the adsorption kinetics of PFSAs by MIEX resin increased with the increasing length of the perfluoroalkyl tail [27]. Therefore, the impact of PFAS structures and resin properties on PFAS adsorption needs to be further investigated. In addition, previous studies have mainly focused on legacy PFASs (e.g., PFOA and PFOS) with emerging alternatives and short PFASs being seldom investigated [28,29]. Therefore, it is essential to perform a comprehensive study of the removal of short and emerging PFASs by resins with different properties.

Moreover, a few studies have evaluated the impact of solution chemical parameters (e.g., pH, dissolved organic matters (DOM), and anions) on PFAS removal by resins [29,30]. For instance, Yang et al. (2018) revealed that pH (5–10) did not significantly influence the adsorption of PFAS onto polyacrylic resin [31]. Conversely, another study reported a greater removal of PFAS by polystyrene resin under pH = 3 as compared to pH = 7 [32]. Additionally, a significant decrease in PFOA adsorption onto polyacrylic resin was observed when pH > 8 [30]. Regarding DOM, Kothawala et al. (2017) reported that 2 mg/L DOM slightly reduced the removal efficiencies (less than 10%) of PFASs by polystyrene resin as compared with DOM-free control [33]. However, another study found that 5 mg/L DOM led to a 24% decrease in PFAS removal by polyacrylic resin [34]. As regards inorganic anions, their impacts on the adsorption of short-chain PFASs and emerging PFASs have rarely been explored [29] but with controversial results being reported. While one study found that 1 mmol/L sulfate had minimal impact on PFOS adsorption onto polyacrylic resin [35], Yang et al. (2018) revealed that the adsorption capacity for PFOA by polyacrylic resin was significantly decreased in the presence of sulfate and carbonate [31]. Fulvic acid (FA) represents over 70% of DOM in surface waters [36]. Moreover, sulfate, carbonate, and bicarbonate are common anions in water [37]. Taken together, the mechanisms of solution chemistry (pH, DOM, and anions) influencing PFASs removal by resins are not fully understood, and a comprehensive study should be conducted.

The main purpose of this study was to investigate the adsorption behavior of ten PFASs (eight legacy PFASs and two alternatives) onto four commercial anionic resins (HPR4700, WA10, S6368, A111S) with different properties. The adsorption kinetics and the competitive behaviors of various PFASs in multisolute aqueous solutions were investigated. Accordingly, the potential adsorption mechanism of different PFASs on multiple resins was also discussed. Additionally, the impact of solution compositions (co-existing DOM and inorganic anions, and pH) on PFASs removal was explored.

## 2. Materials and Methods

### 2.1. Chemicals and Materials

In this study, ten target PFASs (PFBA, PFBS, PFHxA, PFHxS, PFHpA, PFOA, PFOS, PFDA, GenX, and 6:2 FTSA) were purchased from Wellington Laboratories (Guelph, ON, Canada), with their detailed information shown in Tables S1 and S2 in Supplementary Materials (SM). Four types of chloride resins (HPR4700, WA10, S6368, and A111S) were selected. Specifically, HPR4700 (DuPont, Wilmington, NC, USA), WA10 (Mitsubishi Chemical, Tokyo, Japan), S6368 (Lanxess, Cologne, Germany), and A111S (Purolite, King of Prussia, PA, USA) were gel-type strong base resin, gel-type weak base resin, macroreticular strong base resin, and macroreticular weak base resin, respectively. The physical morphology and property of these resins are provided in Figure S1 and Table S3. Fulvic acid (95%) was obtained from Thermo Fisher (Scoresby, North Ryde, Australia). Guaranteed reagents (hydrochloric acid, sodium hydroxide, sodium sulfate, sodium carbonate, and sodium bicarbonate) were all bought from CNW (Shanghai, China). Ultrapure water was produced by the Milli-Q water purification system (Millipore, Boston, MA, USA). Mass spectrometry grade methanol was purchased from Merck (Burlington, MA, USA).

### 2.2. Adsorption Experiments

(i) Removal of PFASs by resins: Adsorption experiments were conducted in 50-mL polypropylene (PP) centrifuge tubes. Specifically, 60 μL of PFASs mixture stock solution (100 mg/L) was added to 30 mL of ultrapure water to reach an initial concentration of 0.2 mg/L for individual PFASs. HCl and NaOH (1 mol/L) were used to adjust the solution pH to 7. After being vortexed, 0.02 g of resin was added into the centrifuge tube. Subsequently, the tubes were oscillated at 25 °C with a shaking speed of 250 rpm for 24 h in an incubator shaker (Benchmark, USA). Finally, 1 mL of sample was collected at the following time intervals: 0, 1, 2, 3, 4, 6, 8, 12 and 24 h for PFAS concentration analysis.

(ii) Effect of DOM and inorganic anions on PFASs adsorption: regarding DOM, the adsorption experiments were still performed in 50 mL of PP tubes, and 30 mL PFAS solutions (0.2 mg/L) were spiked with 500 mg/L of FA stock solution (150, 300, and 1500 μL) to reach FA levels of 0.5, 1, and 5 mg/L in the solution, respectively. Regarding inorganic anions, sodium sulfate, sodium carbonate, and sodium bicarbonate (500 mg/L, 1500 μL) were added to 30 mL of PFAS solutions (0.2 mg/L) to maintain anions levels of 5 mg/L. The subsequent procedures were the same as for the above, part (i).

(iii) Effect of pH on PFASs adsorption: HCl and NaOH (1 mol/L) were used to adjust the pH (3, 5, 7, and 9) of 30 mL of PFAS solutions (0.2 mg/L) in the presence of 1 mg/L FA. The remaining procedures were the same as for the above, part (i).

### 2.3. PFASs Extraction and Determination

One mL of sampled solution was centrifuged at a speed of 13,000 rpm for 10 min in 3K15 centrifuge (Sigma, Darmstadt, Germany), and 100 μL of supernatant was collected and mixed with 400 μL of methanol in a 1-mL centrifuge tube. Next, the mixture solution was filtered through a 0.22 μm inorganic membrane filter into a 1-mL PP vial. The concentrations of the target PFASs were determined using ultrahigh performance liquid chromatography-triple quadrupole mass spectrometry (UPLC-MS/MS) 1290-6460 from Agilent (Agilent Technologies, Palo Alto, USA). Detailed information on instrumental parameters is provided in Text S1, and Tables S1 and S4.

### 2.4. Data Analysis

Nonlinear pseudo-first-order (PFO) and pseudo-second-order (PSO) adsorption kinetics models were used to analyze adsorption mechanisms. These can be expressed as follows [38]:

(1) $${\;(\mathrm{PFO})\;Q}_{t}{=Q}_{e}{(1-e}^{{-k}_{1}t})$$

(2) $$\left(\mathrm{PSO}\right)\;Q_{t}=\frac{Q_{e}{}^{2}K_{2}t}{{1+Q}_{e}K_{2}t}$$

where Q_t_ and Q_e_ (μg/g) are adsorption capacity at the sampling time (h) and equilibrium time (h), respectively; K_1_ (h^−1^) and K_2_ (g/μg.h) represent the adsorption rate coefficient corresponding to PFO and PSO equation, respectively.

Additionally, the experimental adsorption capacity (μg/g) at the sampling time (h) was calculated as follows [39]:

(3) $$Q_{t}=\frac{C_{0}{-C}_{t}}{M}\times \;V$$

where C_0_ and C_t_ (μg/L) are concentrations of PFAS at the initial time and time interval (h), respectively. M (g) is the amount of resin in the solution, and V (L) indicates the volume of the solution.

Moreover, the Weber-Morris IPD model was also used to explore the controlled process of adsorption, which can be expressed as the following equation [40]:

(4) $${\;Q}_{t}{=k}_{\mathrm{id}}\sqrt{t}+C\;$$

where k_id_ is the diffusion rate constant [μg/(g×h^0.5^)], and C denotes the boundary layer thickness. k_id_ and C are the slope and intercept of the linear plot of Q_t_ vs. t^0.5^. If this plot passes through the origin, it suggests that the adsorption process is controlled by intraparticle diffusion. The intercept indicates the effect of external mass transfer.

### 2.5. Quality Assurance/Quality Control (QA/QC)

All adsorption experiments were performed in triplicates, and the blank groups without resins were run under identical conditions. To reduce the possibility of container adsorption and sample contamination, glass containers and Teflon were avoided. A recovery test was conducted to ensure the reliability and accuracy of the extraction method, with values ranging from 95% to 113%. Method limit of quantification (MLQ) and method detection limit (MDL) were obtained based on ten and three times the signal-to-noise (S/N) ratio, respectively. Control and blank samples were performed for each batch to check any background contamination, carryover, and loss of target PFAS, as well as the precision and reliability of the instrument. Full details on recoveries, MLQs, and MDLs are shown in Table S5.

## 3. Results and Discussion

### 3.1. Removal of PFASs by Various Resins

**Figure 1:**
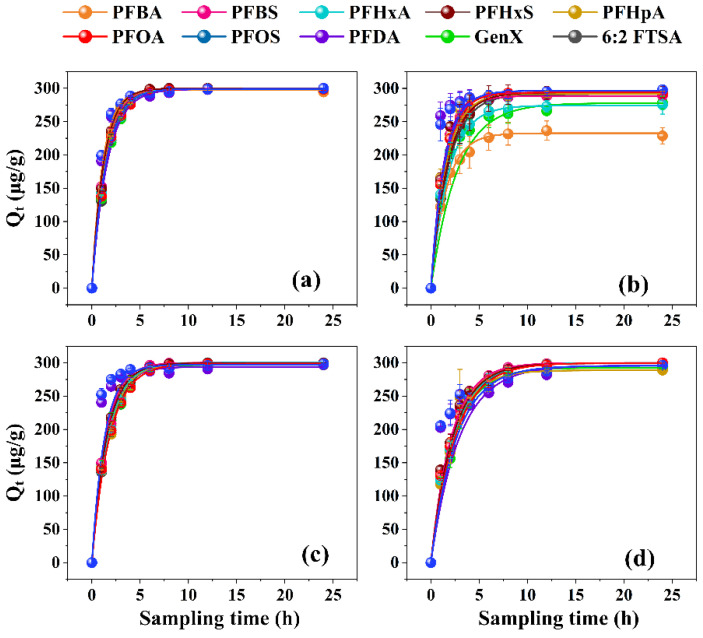
Pseudo-first-order model curve for the adsorption of PFASs onto different resins: (**a**) HPR4700; (**b**) WA10; (**c**) S6368; (**d**) A111S.

**Figure 2:**
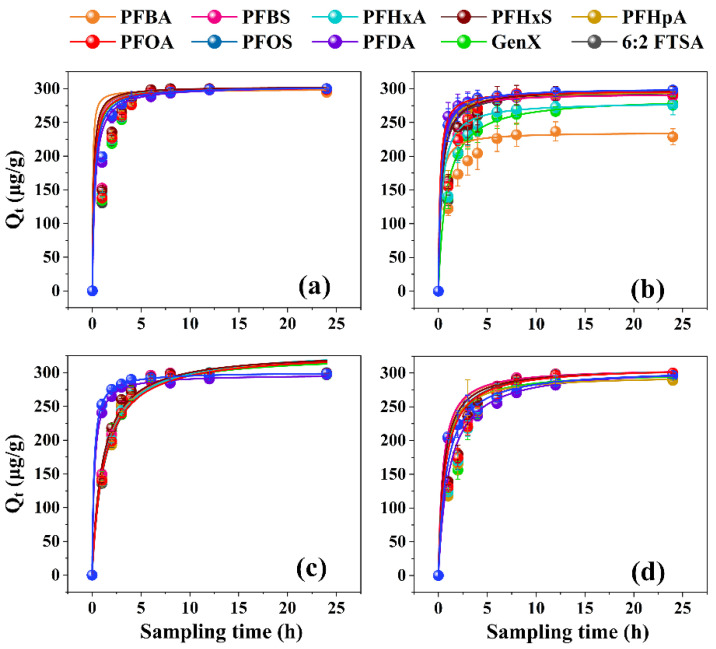
Pseudo-second-order model curve for the adsorption of PFASs onto different resins: (**a**) HPR4700; (**b**) WA10; (**c**) S6368; (**d**) A111S.

As shown in Table S6, there were no significant losses of target PFASs in the blank tests, indicating negligible adsorption of PFASs onto containers. Both the PFO and PSO kinetic models fitted well with the removal of the target PFASs by the four investigated resins (all R^2^ **>** 0.99) (Figure 1 and Figure 2; Tables S7 and S8). The good fitting of the PSO model suggested that the removal of the target PFASs was probably caused by chemical adsorption [41,42]. Nevertheless, the theoretical maximum adsorption capacities obtained by the PFO model (error ≤ 1%) were closer to the experimental values than the PSO model (error ≤ 4%), suggesting that Henry regime adsorption and reaction control might also be involved in the adsorption of trace PFASs onto resins [43,44]. In order to describe the removal performance more accurately, the kinetic parameters obtained by the PFO model were used for data analysis. Overall, the performance of HPR4700, S6368, and A111S was better than WA10 for PFAS removal. Specifically, the capacities of the four resins adsorbing individual PFASs were ranked in the following order: HPR4700 (297~300 μg/g) ≈ S6368 (294~300 μg/g) ≈ A111S (289~300 μg/g) > WA10 (233~297 μg/g). In addition, the adsorption rate coefficients of HPR4700 (0.62~0.89) and S6368 (0.55~0.76) were higher than WA10 (0.37~0.76) and A111S (0.34~0.47). Therefore, it seems that the difference in the functional group could affect the capability of resins to remove PFAS, as the quaternary ammonium group was present on the surface of HPR4700 and S6368, while tertiary amine was the functional group for WA10 and A111S (Table S3). Since previous studies have found that the anion-exchange interactions between anionic pollutants and strong base resin were stronger than those with weak base resin [45,46], the better performance of HPR4700 and S6368 observed here could be attributed to their stronger anion-exchange interactions with anionic PFASs as compared to WA10 and A111S. Further, even though WA10 and A111S had the same functional group (tertiary amine), it seems that A111S could remove PFASs more efficiently than WA10, possibly because of the difference in resin morphology. For example, A111S is a macroreticular resin, whereas WA10 is a gel-state resin. According to previous studies, macroreticular resin had a permanent pore structure with a large pore size, while gel-type resin needed to swell in water and then generate an inner pore with a small size [47,48]. Therefore, the target PFASs were likely to diffuse into the pore spaces of A111S more easily than those of WA10, thereby generating higher adsorption capacities on A111S than on WA10. Moreover, the matrices of A111S and WA10 were polystyrene and polyacrylic, respectively, which might be another influencing factor, as previous studies have shown that the hydrophobicity of the resin backbone could facilitate the removal process for organic pollutants [49,50]. Because the polystyrene structure was more hydrophobic than the polyacrylic structure for its aromatic ring, the hydrophobic interactions between A111S and the target PFASs might be stronger than those with WA10. Similarly, a previous study has found that the hydrophobic A532E exhibited higher removal efficiency for PFASs compared to hydrophilic A600E [25].

**Figure 3:**
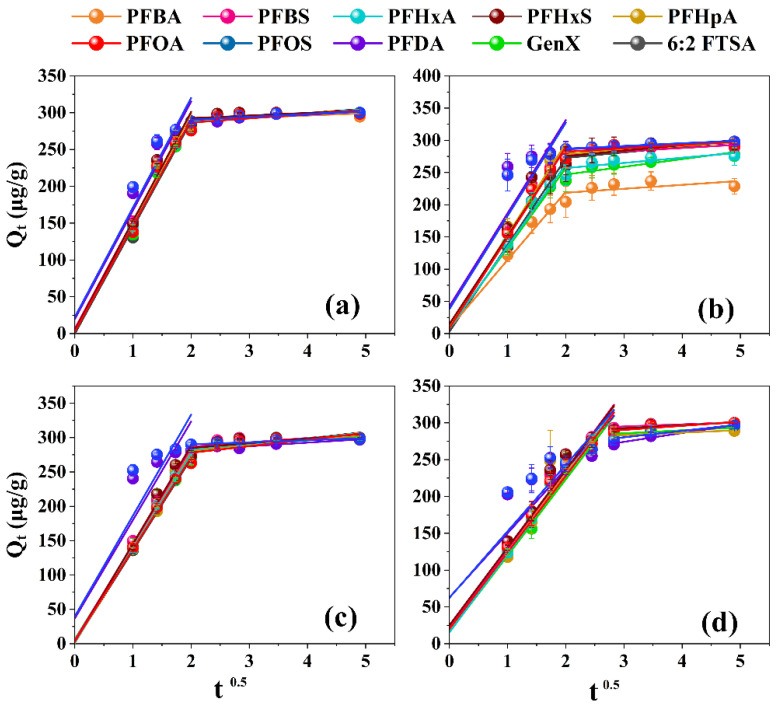
Weber-Morris IPD model curve for the adsorption of PFASs onto different resins: (**a**) HPR4700; (**b**) WA10; (**c**) S6368; (**d**) A111S.

It was clear that the PFAS structure would also influence their removal efficiencies (Figure S2). Noteworthily, while preferential adsorption for PFAS with longer C-F chains occurred for WA10, the adsorption removal of PFAS was negatively correlated with their C-F backbone lengths for S6368 and A111S. As regards HPR4700, the adsorption capacities for PFOA (CF_7_), PFOS (CF_8_), and PFDA (CF_9_) were also less than shorter PFASs. As shown in Table S3, the moisture content of WA10 (63~69%) was generally higher than those of the remaining resins. Since higher moisture contents indicated a smaller crosslinking degree and higher porosity of resins [51,52], WA10 might have more pore spaces and channels. This finding means that the internal diffusion of PFAS in WA10 would be easier than in other resins. For the remaining three selected resins, lower porosity was probably not favorable to remove PFAS with a longer backbone due to the steric effects [53]. There was no significant difference in the adsorption capacity between PFCA and PFSA with the same C-F skeleton. Compared with legacy PFOS, the alternative 6:2 FTSA was more easily removed, perhaps due to the weaker steric hindrance of 6:2 FTSA leading to its quicker internal mass transfer. Nevertheless, the emerging GenX was more difficult to remove than legacy PFOA in most cases. Apparently, PFOA (CF_7_) was much more hydrophobic than GenX (CF_4_), thus making the removal of PFOA easier. The fitted results of the Weber-Morris IPD model revealed the intraparticle diffusion effect during the adsorption (Figure 3; Table S9). For HPR4700, WA10, and S6368, the fitting line almost passed through the origin for most target PFASs except for PFOS and PFDA. On the contrary, for A111S, none of the fitting curves passed through the origin. As the IPD plot with a lower intercept indicated that the adsorptive process was regulated by intraparticle diffusion [40,54], it could be speculated that the adsorption of PFASs by HPR4700, WA10, and S6368 was dominated by intraparticle diffusion, while the removal of PFASs by A111S was controlled by both film and intraparticle diffusion. Therefore, intraparticle diffusion was apparently more important than film diffusion during the adsorption process of PFAS by resins, a conclusion in agreement with the findings of a previous paper [50].

### 3.2. Effect of FA and Inorganic Anions on PFASs Adsorption

**Figure 4:**
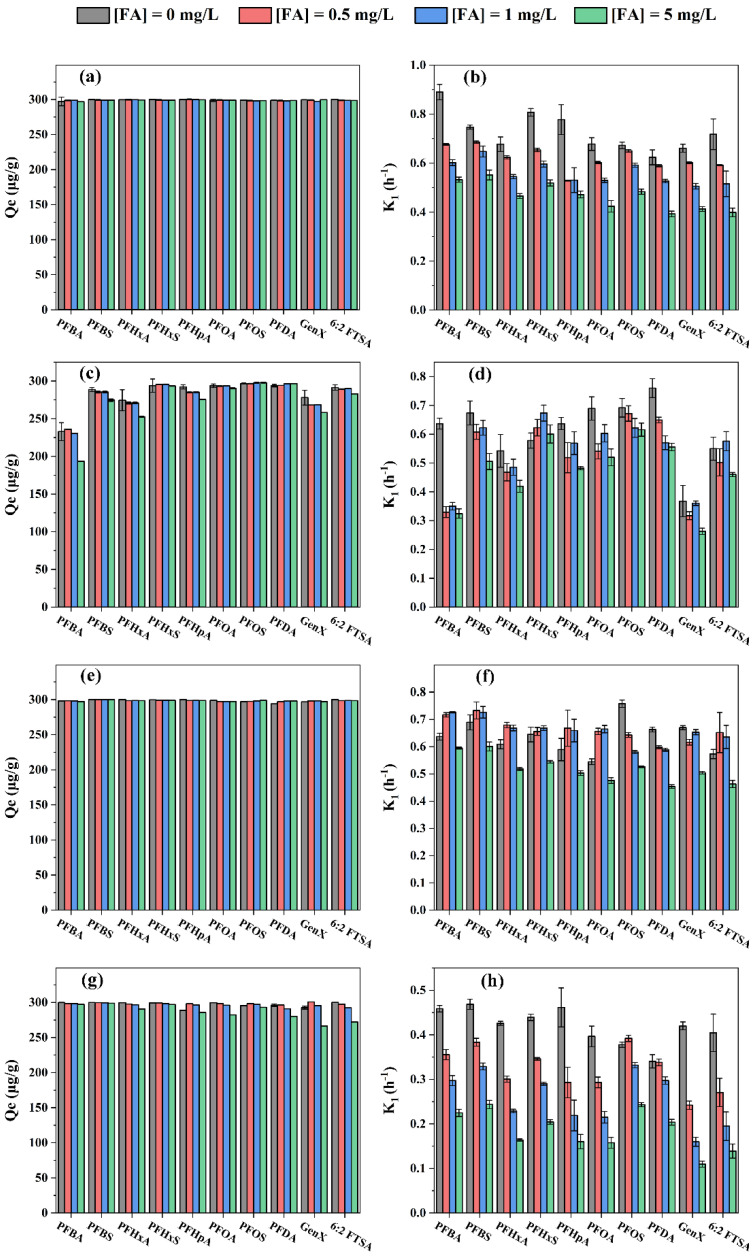
Adsorptive removal of PFASs by various resins in the presence of FA: (**a**,**b**) HPR4700; (**c**,**d**) WA10; (**e**,**f**) S6368; (**g**,**h**) A111S.

The adsorption removal of PFASs varied among the four resins in the presence of FA (Figure 4). Specifically, the adsorption capacity for all target PFASs on HPR4700 and S6368 was fairly consistent over the FA concentrations ranging from 0~5 mg/L. By contrast, slight reductions in adsorption capacity occurred on WA10 and A111S. The adsorption capacities of short-chain PFASs on WA10 decreased obviously in the presence of 5 mg/L FA. For example, adsorption capacities of PFBA, PFBS, GenX, PFHxA, and PFHpA decreased from 233 to 194 μg/g, 289 to 275 μg/g, 278 to 258 μg/g, 274 to 253 μg/g, and 292 to 276 μg/g, respectively. Similarly, for A111S, adsorption capacities of GenX, 6:2 FTSA, PFOA, and PFDA decreased from 293 to 266 μg/g, 300 to 272 μg/g, 300 to 282 μg/g, and 296 to 280 μg/g, respectively. Therefore, the inhibition of PFAS removal caused by FA was less obvious for strong base resin than for weak base resin. Previous studies have suggested that the coexisting DOM could compete with the target pollutants for adsorption sites, thereby hindering the removal of the target substances [55,56]. In addition, pollutants with high concentrations might contact an adsorbent more easily than trace substances [57]. As the adsorption sites on WA10 and A111S might be insufficient for both PFASs and FA, FA would likely occupy adsorption sites more rapidly for their higher content, thus creating fewer sites available for the trace PFASs. Moreover, as strong base resin showed a stronger anion-exchange interaction with PFAS than weak base resin, trace PFAS might still maintain intensive interaction with strong base resin in the presence of FA. The coexisting FA not only reduced the adsorption capacity but also affected the adsorption speed (Figure 4). For HPR4700 and A111S, the rate coefficient K_1_ decreased gradually with the increasing FA concentrations. The K_1_ of PFASs removal by HPR4700 under 0, 0.5, 1, and 5 mg/L FA were in the range of 0.62~0.89, 0.53~0.69, 0.51~0.65, and 0.39~0.55, respectively. Therefore, FA could obviously inhibit the adsorption of PFASs on HPR4700 through adsorption speed rather than adsorption capacity, a fact that might be related to the sufficient adsorption sites on HPR4700. Likewise, K_1_ of PFASs adsorption by A111S under 0, 0.5, 1, and 5 mg/L FA were in the range of 0.34~0.47, 0.24~0.39, 0.16~0.33, and 0.11~0.24. As most DOM carried negative charges in water due to their acid functional groups, electrostatic repulsions from resins to anionic pollutants would be enhanced with increasing DOM concentrations [58,59]. Moreover, as both HPR4700 and A111S had small pore spaces, more negative FA loaded in resin might decelerate the adsorption of the target PFASs. For WA10, the K_1_ values also generally decreased, but the rate coefficients of PFASs under 1 mg/L FA were higher than those of 0.5 mg/L and 5 mg/L, except for PFOS and PFDA. Previous studies have reported that co-adsorption was likely to occur between PFAS and aromatic DOM, a factor that might promote the removal process [60,61]. Therefore, 5 mg/L FA might block more sites while co-adsorption between PFAS and FA might not occur easily under 0.5 mg/L FA due to the low FA content. Additionally, the aromatic FA might enhance the hydrophobicity of WA10 matrix, also facilitating the removal of short-chain PFAS [62]. Surprisingly, S6368 could remove most target PFASs faster in the presence of 0.5 and 1 mg/L FA than that in pure water. Because S6368 is a strong base resin with macropore, pore blockage in S6368 might not be obvious, while hydrophobic FA was likely to enhance the hydrophobic interaction between PFASs and S6368, resulting in more rapid adsorption of PFASs.

**Figure 5:**
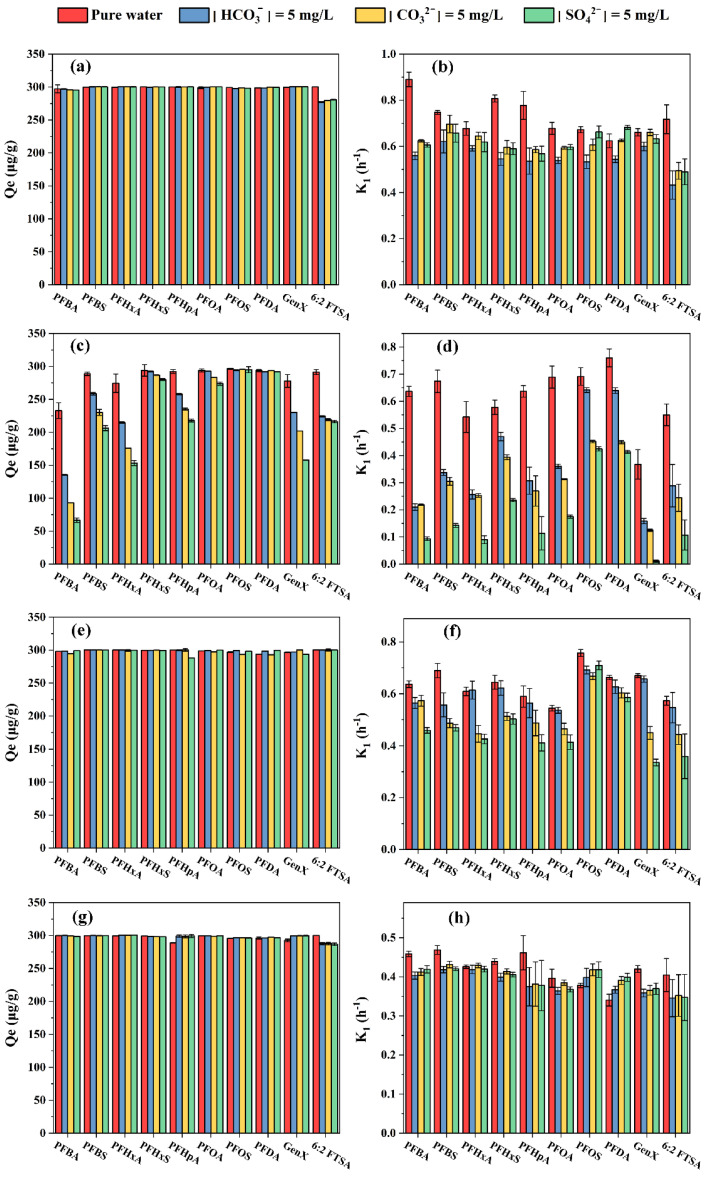
Adsorptive removal of PFASs by various resins in the presence of inorganic anions: (**a**,**b**) HPR4700; (**c**,**d**) WA10; (**e**,**f**) S6368; (**g**,**h**) A111S.

The adsorption capacity of most PFASs by HPR4700, S6368, and A111S did not decrease obviously in the presence of different anions (Figure 5). However, co-existing anions inhibited the removal of short-chain PFASs and two alternatives by WA10. For example, the adsorption capacity of PFBA by WA10 decreased from 233 to 67 μg/g, and PFBS, PFHxA, GenX, and 6:2 FTSA showed similar values, decreasing from 289 to 206 μg/g, 274 to 153 μg/g, 278 to 152 μg/g, and 291 to 216 μg/g in the presence of sulfate, carbonate, and bicarbonate. Moreover, the inhibition was generally ranked in the following order: sulfate > carbonate > bicarbonate. Inorganic anions in water might compete with target pollutants for adsorption sites, and anionic pollutants with sufficient charges were more easily adsorbed [63,64]. Anion-exchange interaction between WA10 and PFAS was weak, and adsorption sites on WA10 might be insufficient to simultaneously adsorb PFASs and the competitive anions. The electrostatic interactions between inorganic anions and resins were stronger than those with anionic PFASs, thus making the removal of inorganic anions by WA10 preferable. Sulfate and carbonate demonstrated greater charges, so they were likely to occupy adsorption sites more easily than bicarbonate and shorter PFASs. The steric volume of sulfate is larger than carbonate, making its interaction with resin more favorable. Noteworthily, the performance of A111S for PFAS removal in the presence of competitive anions was much better than that of WA10, a result which implies that the macroreticular weak base resin is more promising for removing trace pollutants than gel weak base resin. Similar to FA, the competitive anions would also retard PFAS adsorption speed in most cases (Figure 5). Therefore, it could be assumed that anions would restrict the PFAS adsorption by HPR4700, S6368, and A111S, but by adsorption speed rather than removal efficiency. This result was probably related to the adequate adsorption sites for both PFASs and anions on HPR4700, S6368, and A111S. Interestingly, A111S would adsorb PFOS and PFDA more rapidly in the presence of anions. Previous studies suggested that anions adsorbed onto adsorbent surfaces would raise electrostatic repulsion to anionic pollutants, and short-chain PFASs were mainly adsorbed by electrostatic interaction [19,65]. Therefore, sulfate, carbonate, and bicarbonate adsorbed on A111S enhanced the electrostatic repulsion to short-chain PFASs and hinder their adsorption. However, PFOS and PFDA might be able to overcome the electrostatic repulsion through their longer hydrophobic backbones, giving them an advantage in adsorption competition.

### 3.3. Effect of pH on PFASs Adsorption

**Figure 6:**
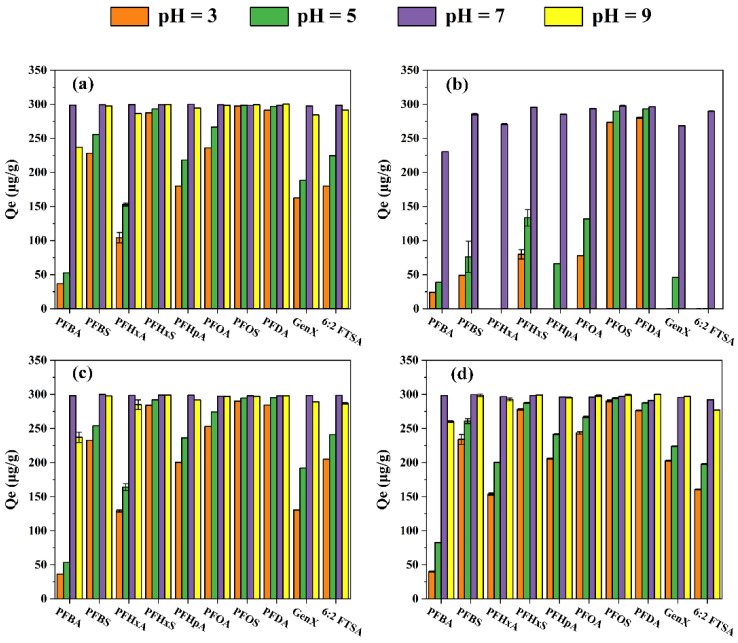
Adsorptive removal of PFASs by various resins under different pH conditions: (**a**) HPR4700; (**b**) WA10; (**c**) S6368; (**d**) A111S.

The pH influenced the PFAS adsorption on the four resins (Figure 6). Generally, the adsorption capacities of all PFASs by HPR4700, S6368, and A111S under different pH conditions were ranked in order: pH = 7 and 9 > pH = 5 > pH = 3. Specifically, adsorption capacities of PFASs by HPR4700 at pH = 3, 5, 7, and 9 were in the range of 37~297 μg/g, 53~298 μg/g, 298~300 μg/g, and 237~300 μg/g, respectively. S6368 and A111S also presented similar trends corresponding to pH = 3, 5, 7, and 9. Moreover, the removal efficiencies of PFASs by HPR4700, S6368, and A111S at pH = 7 and 9 were similar, except for PFBA. As is well known, the removal of anionic contaminants (A^−^) by chloride resins relies on the following processes:

[R_3_ N^+^]Cl^−^ + A^−^ = [R_3_N^+^]A^−^ + Cl^−^ (strong base)

[R_2_ N]Cl^−^ + H^+^ + A^−^ = [R_2_NH^+^]A^−^ + Cl^−^ (weak base)

Therefore, the dissociation of PFAS could be inhibited under an acidic medium, resulting in the reduction of adsorption capacity due to the weakened anion-exchange interaction [66,67]. When the solution contained sufficient hydroxide ions, resin might adsorb them and generate electrostatic repulsion to short-chain PFBA, thus reducing its adsorption capacity. Obviously, pH had a greater impact on WA10 than on other resins. For WA10, pH = 7 was favorable for removing all target PFASs, pH = 3 or 5 could only achieve high adsorption capacities for PFOS and PFDA, and no removal was observed for any PFASs at pH = 9. For instance, the adsorption capacity for PFBA achieved 24 μg/g, 39 μg/g, 230 μg/g, and 0 μg/g under pH = 3, 5, 7, 9, respectively. In addition, 6:2 FTSA and PFHxA could only be removed at pH = 7, and PFOS and PFDA could achieve higher adsorption capacities at pH = 3, 5, and 7 (all ≥ 273 μg/g). According to the ion exchange reaction of the weak base resin, the protons were essential to the adsorption of contaminants by the weak base resin [68,69]. Consequently, the higher pH might extremely hinder the ion exchange reaction of PFAS by WA10. One possible reason was the fewer protons in an alkaline solution. In addition, the polyamine groups of WA10 would change to the base form at high pH, thus resulting in a reduction of PFAS adsorption capacity due to the weakened ability for anion exchange [21]. Nevertheless, WA10 and A111S are both weak base resins, but the removal efficiency of A111S was much better than WA10 under pH = 9, which suggests that the performance of gel weak base resin was extremely sensitive to pH. The conversion of polyamine groups on weak base resin to base forms reduced the number of efficient adsorption sites so that the adsorption competition between PFAS and FA would become more obvious and FA might be adsorbed preferentially due to its high concentration. Thus, the limited adsorption sites on WA10 were occupied by the competitive FA completely at pH = 9, but A111S still possessed enough adsorption sites for PFAS removal. This factor might be the reason for their different performance at pH = 9. Therefore, the gel weak base resin was not well suited for removing trace PFAS under the alkaline medium. In agreement with these results, a previous study also reported that the performance of gel weak base resin IRA67 could deteriorate with increasing pH [21].

## 4. Conclusions

A number of resins evaluated in this study enabled the removal of PFAS from aqueous solutions. Generally, the performance of strong base resins was better than that of weak base resins for PFAS removal. The removal efficiency of PFASs by gel-type weak base resins was inferior to macroreticular weak base resins, especially for short-chain PFASs. Moreover, among the four investigated resins, only the gel-type weak base resin showed relative preferential adsorption for long-chain to short-chain PFASs. Chemical and Henry regime adsorption or reaction control might be involved in the PFAS removal process. Furthermore, PFASs adsorption by resins was probably controlled by intraparticle diffusion in most cases. Both FA and inorganic anions could inhibit PFAS removal, and the inhibition was most obvious for the gel-type weak base resins. Gel-type strong base resins and macroreticular base resins more efficiently removed PFAS under a neutral and alkaline medium than an acidic medium. However, the performance of the gel-type weak base resin was likely to be deteriorated in an extremely alkaline medium, leading to negligible PFAS removal.

## Data Availability

The data presented in this study are available on request from the corresponding author.

## Supplementary Materials

The following supporting information can be downloaded at: https://www.mdpi.com/article/10.3390/toxics11020161/s1, Text S1; Figure S1: The physical morphologies of various resins used in the present study; Figure S2: The linear correlation between the C-F chain length and the adsorption capacity of PFASs on various resins; Table S1: The analyte formula, manufacturer, acronym, and optimum LC-MS/MS parameters for multiple reaction monitoring (MRM) acquisition conditions of individual PFASs; Table S2: The acidity coefficient and chemical structures of ten PFASs; Table S3: The property parameters of the different resins; Table S4: The gradient elution of the mobile phase 2 mM ammonium acetate (A) and methanol (B); Table S5: MDLs, MLQs and Recoveries of PFASs in the sample solution (n=3); Table S6: The fluctuation range of C/C_0_ of each PFAS after 24 hours without adsorbent as a blank group; Table S7: Kinetic parameters of the pseudo-first-order model for adsorption of PFASs on different resins in pure aquatic system; Table S8: Kinetic parameters of the pseudo-second-order model for adsorption of PFASs on different resins in pure aquatic system; Table S9: Kinetic parameters of the Weber–Morris IPD model for adsorption of PFASs on different resins in pure aquatic system.
