# Supplementary material for: Insights into the Understanding of Adsorption Behaviors of Legacy and Emerging Per- and Polyfluoroalkyl Substances (PFASs) on Various Anion-Exchange Resins
Source: Toxics. 2023 Feb 9;11(2):161. doi: 10.3390/toxics11020161 (PMC9961606; doi:10.3390/toxics11020161)
Supplement: Supplementary file 1 [file toxics-11-00161-s001.zip › toxics-2162817-supplementary.pdf]

Supplementary Material

Toward systematic understanding of adsorption behavior of legacy and emerging per-and polyfluoroalkyl substances (PFASs) on various anion-exchange resins

Hong-Ming Tan [1,2], Chang-Gui Pan [1,3,*], Chao Yin [1] and Kefu Yu [1,2,*]

[1] Guangxi Laboratory on the Study of Coral Reefs in the South China Sea, School of Marine Sciences, Guangxi University, Nanning 530004, China

[2] School of Resources, Environment and Materials, Guangxi University, Nanning 530004, China

[3] Southern Marine Science and Engineering Guangdong Laboratory, Zhuhai 519000, China

[*] Correspondence: panchanggui@gxu.edu.cn (C.-G.P.); kefuyu@scsio.ac.cn (K.Y.)

Text S1

Eclipse Plus C18 column (2.1 mm i. d. × 50 mm length, 1.8 μm; Agilent Technologies, USA) was used to achieve chromatographic separation. The mobile phase consisted 2 mM ammonium acetate in water (A) and methanol (B) with the flow rate of 0.25 mL/min. The analysis was carried out with Jetstream electrospray ionization (ESI) source in negative mode.

Table S1. The analyte formula, manufacturer, acronym, and optimum LC-MS/MS parameters for multiple reaction monitoring (MRM) acquisition conditions of individual PFASs

Compound	Acronym	Formula	MS/MS mass transition	Fragmentor	Collision energy	Manufacturer
Perfluorobutanoic acid	PFBA	C_3F_7COOH	213-168.8[a]	65	5	Wellington laboratories,
Perfluorobutane sulfonic acid	PFBS	$C_4F_9SO_3H$	299-79.9[a]/98.9[b]	145	41/29	Wellington laboratories,
Perfluorohexanoic acid	PFHxA	$C_5F_{11}COOH$	313-118.9[a]/268.8[b]	70	1/13	Wellington laboratories,
Perfluorohexane sulfonic acid	PFHxS	$C_6F_{13}SO_3H$	398.9-79.9[a]/98.9[b]	165	53/37	Wellington laboratories,
Perfluoroheptanoic acid	PFHpA	$C_6F_{13}COOH$	363-168.8[a]/318.8[b]	70	1/9	Wellington laboratories,
Perfluorooctanoic acid	PFOA	$C_7F_{15}COOH$	413-368.7[a]/168.9[b]	80	1/9	Wellington laboratories,
Perfluorooctane sulfonic acid	PFOS	$C_8F_{17}SO_3H$	498.9-79.9[a]/98.9[b]	190	69/45	Wellington laboratories,
Perfluorodecanoic acid	PFDA	$C_9F_{19}COOH$	513-468.7[a]/218.8[b]	85	5/13	Wellington laboratories,
6:2 Fluorotelomer sulfonate	6:2 FTSA	$CF_3(CF_2)_5(CH_2)_2SO_3H$	427-407 [a]/81 [b]	135	24/43	Wellington laboratories,
Hexafluoropropylene oxide dimer acid	GenX	$C_3F_7OCF(CF_3)COOH$	329-285 [a]/169 [b]	39	1/11	Wellington laboratories,

[a] The product ion used for quantification;

[b] The product ion used for qualification;

Chemical	pKa (25°C)	Chemical structure
PFBA	1.07 [1]	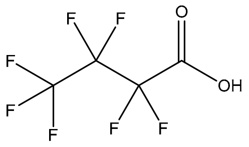
PFBS	-3.31 [1]	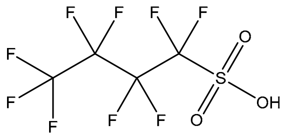
PFHxA	-0.78 [2]	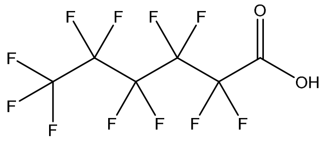
PFHxS	0.14 [3]	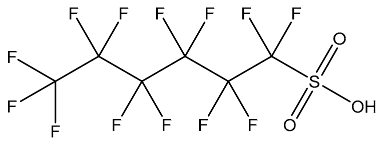
PFHpA	-2.29 [1]	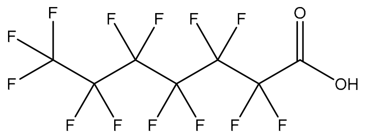
PFOA	-0.5-4.2 [1]	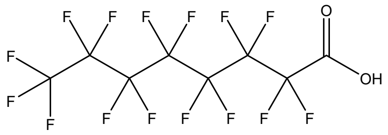
PFOS	-3.27 [2]	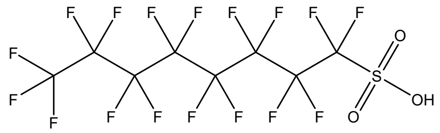
PFDA	-5.2 [1]	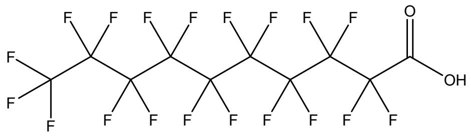
6:2 FTSA	<1.0 [1]	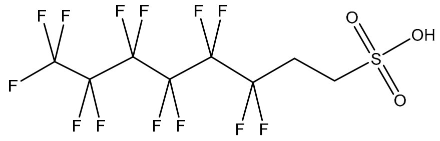
GenX	2.84 [2]	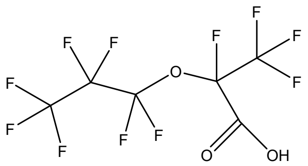

Table S2. The acidity coefficient and chemical structures of ten PFASs

Table S3. The property parameters of the different resins.

Type of resin	Matrix	Functional group	Exchange capacity (eq/L)	Average particle size (μm)	Moisture content (%)
HPR4700	Polystyrene	Quaternary ammonium	1.35	550	49-55
WA10	Polyacrylic	Tertiary amine	1.2	510	63-69
S6368	Polystyrene	Quaternary ammonium	1.1	610	58-64
A111S	Polystyrene	Tertiary amine	1.7	712	56-62

Table S4. The gradient elution of the mobile phase 2 mM ammonium acetate (A) and methanol (B)

Time (minutes)	Mobile phase A (%)	Mobile phase B (%)
0	90	10
0.1	65	35
7	45	55
17	5	95
18	5	95
20	90	10

Table S5. MDLs, MLQs and Recoveries of PFASs in the sample solution (n = 3)

Compound	MDL (μg/L)	MLQ (μg/L)	Recovery (%)
PFBA	0.064	0.213	102
PFBS	0.058	0.194	97
PFHxA	0.018	0.059	103
PFHxS	0.149	0.497	110
PFHpA	0.048	0.161	95
PFOA	0.009	0.030	113
PFOS	0.275	0.917	102
PFDA	0.006	0.018	109
GenX	0.551	1.836	96
6:2 FTSA	0.008	0.026	101

MDL: method detection limits; MLQ: method limits of quantification

Table S6. The fluctuation range of C/C_0 of each PFAS after 24 hours without adsorbent as a blank group

PFAS	C/C_0
PFBA	0.980-1.017
PFBS	1.001-1.039
PFHxA	1.019-1.051
PFHxS	0.997-1.037
PFHpA	1.027-1.071
PFOA	1.045-1.090
PFOS	0.919-1.104
PFDA	0.956-1.099
GenX	0.959-1.021
6:2 FTSA	1.033-1.067

Table S7. Kinetic parameters of the pseudo-first-order model for adsorption of PFASs on different resins in pure aquatic system

Compound	Type of resin	K_1 (h^{-1})	Q_e (μg/g)	R^2	∣△Q∣
PFBA	HPR4700	0.89	297.28	0.99	2.67
	WA10	0.64	232.80	0.99	4.08
	S6368	0.64	298.06	0.99	0.28
	A111S	0.46	299.88	0.99	0.05
PFBS	HPR4700	0.75	299.95	0.99	0.03
	WA10	0.67	288.71	0.99	0.56
	S6368	0.69	300.21	0.99	0.21
	A111S	0.47	299.82	0.99	0.17
GenX	HPR4700	0.66	299.60	0.99	0.02
	WA10	0.37	277.92	0.99	0.42
	S6368	0.67	296.78	0.99	0.06
	A111S	0.42	292.53	0.99	0.17
PFHxA	HPR4700	0.68	299.60	0.99	0.06
	WA10	0.54	274.39	0.99	0.96
	S6368	0.61	300.17	0.99	0.17
	A111S	0.43	299.63	0.99	0.13
PFHxS	HPR4700	0.81	300.17	0.99	0.17
	WA10	0.58	293.91	0.99	0.75
	S6368	0.65	299.68	0.99	0.06
	A111S	0.43	299.27	0.99	0.39
6:2 FTSA	HPR4700	0.72	300.26	0.99	0.26
	WA10	0.55	291.40	0.99	0.58
	S6368	0.57	300.19	0.99	0.19
	A111S	0.41	299.98	0.99	0.02
PFHpA	HPR4700	0.78	300.14	0.99	0.14
	WA10	0.64	292.21	0.99	0.82
	S6368	0.59	299.98	0.99	0.02
	A111S	0.46	288.73	0.99	0.32
PFOA	HPR4700	0.68	298.81	0.99	0.31
	WA10	0.69	293.84	0.99	1.23
	S6368	0.55	298.85	0.99	0.14
	A111S	0.40	299.69	0.99	0.18
PFOS	HPR4700	0.67	299.20	0.99	0.76
	WA10	0.69	296.51	0.99	1.46
	S6368	0.76	297.01	0.99	1.52
	A111S	0.38	295.51	0.99	0.61
PFDA	HPR4700	0.62	298.81	0.99	0.79
	WA10	0.76	293.72	0.99	1.45
	S6368	0.66	293.89	0.99	3.26
	A111S	0.34	296.01	0.99	0.35

Note：∣△Q∣ is the difference value between the adsorption capacity fitted by kinetic model and the experimental value.

Table S8. Kinetic parameters of the pseudo-second-order model for adsorption of PFASs on different resins in pure aquatic system

Compound	Type of resin	K_2 (g/μg.h)	Q_e (μg/g)	R^2	∣△Q∣
PFBA	HPR4700	0.07	298.50	0.99	3.89
	WA10	0.02	235.46	0.99	6.74
	S6368	0.02	302.27	0.99	3.93
	A111S	0.01	307.61	0.99	7.77
PFBS	HPR4700	0.02	303.35	0.99	3.44
	WA10	0.02	293.08	0.99	3.81
	S6368	0.02	303.61	0.99	3.61
	A111S	0.01	307.35	0.99	7.36
GenX	HPR4700	0.02	303.60	0.99	4.02
	WA10	0.01	287.58	0.99	9.24
	S6368	0.02	300.37	0.99	3.65
	A111S	0.01	301.59	0.99	8.89
PFHxA	HPR4700	0.02	303.41	0.99	3.74
	WA10	0.01	280.40	0.99	5.05
	S6368	0.02	304.54	0.99	4.54
	A111S	0.01	308.34	0.99	8.57
PFHxS	HPR4700	0.03	303.14	0.99	3.14
	WA10	0.01	299.78	0.99	5.12
	S6368	0.02	304.01	0.99	4.39
	A111S	0.01	307.92	0.99	8.25
6:2 FTSA	HPR4700	0.02	303.52	0.99	3.52
	WA10	0.01	297.11	0.99	5.14
	S6368	0.01	304.90	0.99	4.90
	A111S	0.01	310.05	0.99	10.05
PFHpA	HPR4700	0.02	303.33	0.99	3.33
	WA10	0.01	297.03	0.99	3.99
	S6368	0.01	304.77	0.99	4.81
	A111S	0.01	296.74	0.99	7.69
PFOA	HPR4700	0.02	302.52	0.99	4.02
	WA10	0.02	297.13	0.99	4.52
	S6368	0.01	304.37	0.99	5.38
	A111S	0.01	309.93	0.99	10.06
PFOS	HPR4700	0.01	303.57	0.99	3.61
	WA10	0.01	301.08	0.99	3.11
	S6368	0.02	300.63	0.99	2.10
	A111S	0.01	306.53	0.99	10.41
PFDA	HPR4700	0.01	303.97	0.99	4.38
	WA10	0.02	297.29	0.99	2.12
	S6368	0.01	300.36	0.99	3.21
	A111S	0.01	309.71	0.99	13.35

Note：∣△Q∣ is the difference value between the adsorption capacity fitted by kinetic model and the experimental value.

Table S9. Kinetic parameters of the Weber–Morris IPD model for adsorption of PFASs on different resins in pure aquatic system

Compound	Type of resin	K_{id}	C	R^2
PFBA	HPR4700	146.02	5.73	0.98
	WA10	105.83	8.50	0.97
	S6368	139.12	3.26	0.99
	A111S	107.53	17.73	0.96
PFBS	HPR4700	146.62	6.43	0.98
	WA10	140.23	11.15	0.97
	S6368	139.84	4.96	0.99
	A111S	107.21	21.35	0.96
GenX	HPR4700	144.61	0.28	0.99
	WA10	123.95	8.98	0.97
	S6368	136.19	6.47	0.99
	A111S	103.53	16.10	0.96
PFHxA	HPR4700	143.71	3.75	0.98
	WA10	127.59	7.76	0.98
	S6368	137.04	1.81	0.99
	A111S	106.64	15.31	0.97
PFHxS	HPR4700	148.15	4.89	0.98
	WA10	136.61	14.97	0.94
	S6368	142.85	2.63	0.99
	A111S	105.42	25.02	0.94
6:2 FTSA	HPR4700	144.39	0.39	0.98
	WA10	134.45	2.96	0.99
	S6368	133.84	1.84	0.99
	A111S	103.97	19.78	0.96
PFHpA	HPR4700	148.33	2.97	0.98
	WA10	141.73	11.71	0.97
	S6368	137.38	0.97	0.99
	A111S	104.19	18.84	0.92
PFOA	HPR4700	144.38	2.32	0.98
	WA10	140.19	8.57	0.98
	S6368	134.14	3.26	0.99
	A111S	103.94	20.11	0.96
PFOS	HPR4700	148.96	22.07	0.93
	WA10	144.63	38.05	0.91
	S6368	146.78	39.67	0.91
	A111S	90.22	62.43	0.89
PFDA	HPR4700	147.79	19.59	0.94
	WA10	144.47	42.54	0.89
	S6368	143.27	36.74	0.89
	A111S	87.26	62.84	0.88

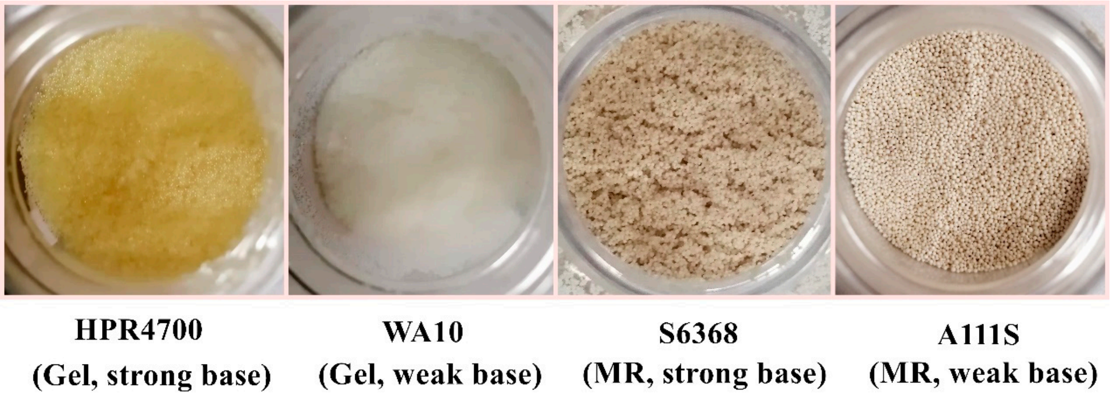

Figure S1. The physical morphologies of various resins used in the present study.

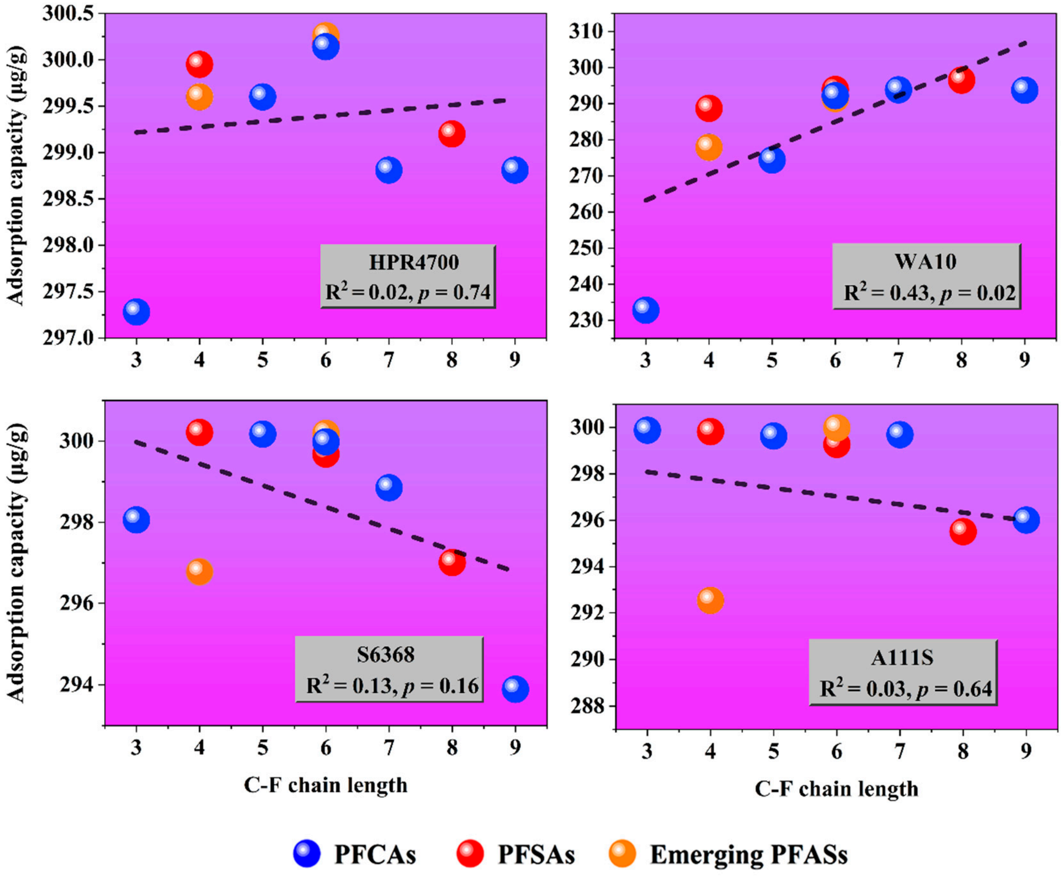

Figure S2. The linear correlation between the C-F chain length and the adsorption capacity of PFASs on various resins.

References

1.Gagliano, E., Sgroi, M., Falciglia, P.P., Vagliasindi, F.G.A. and Roccaro, P. Removal of poly- and perfluoroalkyl substances (PFAS) from water by adsorption: Role of PFAS chain length, effect of organic matter and challenges in adsorbent regeneration. *Water Res*, **2020**, *171*, 115381.

2.Boyer, T.H.; Fang, Y.; Ellis, A.; Dietz, R.; Choi, Y.J.; Schaefer, C.E.; Higgins, C.P.; Strathmann, T.J. Anion exchange resin removal of per- and polyfluoroalkyl substances (PFAS) from impacted water: A critical review. *Water Res*, **2021**, *200*, 117244.

3.Ateia, M.; Maroli, A.; Tharayil, N.; Karanfil, T. The overlooked short- and ultrashort-chain poly- and perfluorinated substances: A review. *Chemosphere*, **2019**, *220*, 866–882.
